# Gender Differences in the Levels of Periodontal Destruction, Behavioral Risk Factors and Systemic Oxidative Stress in Ischemic Stroke Patients: A Cohort Pilot Study

**DOI:** 10.3390/jcm9061744

**Published:** 2020-06-04

**Authors:** Ioana Stănescu, Adriana Elena Bulboacă, Iulia Cristina Micu, Sorana D. Bolboacă, Dana Gabriela Feștilă, Angelo C. Bulboacă, Gyorgy Bodizs, Gabriela Dogaru, Paul Mihai Boarescu, Aurel Popa-Wagner, Alexandra Roman

**Affiliations:** 1 Department of Neurology, Iuliu Hațieganu University of Medicine and Pharmacy Cluj-Napoca, Louis Pasteur Str., no. 6, 400349 Cluj-Napoca, Romania; ioanastane@yahoo.com (I.S.); angelo.bulboaca@yahoo.com (A.C.B.); 2 Department of Pathophysiology, Iuliu Hațieganu University of Medicine and Pharmacy Cluj-Napoca, Louis Pasteur Str., no. 6, 400349 Cluj-Napoca, Romania; boarescu.paul@umfcluj.ro; 3 Department of Periodontology, Faculty of Dental Medicine, IuliuHaţieganu University of Medicine and Pharmacy, Victor Babeş Str., no. 15, 400012 Cluj-Napoca, Romania; i.cristina.micu@gmail.com (I.C.M.); veve_alexandra@yahoo.com (A.R.); 4 Department of Medical Informatics and Biostatistics, IuliuHațieganu University of Medicine and Pharmacy Cluj-Napoca, Louis Pasteur Str., no. 6, 400349 Cluj-Napoca, Romania; 5 Department of Orthodontics, Iuliu Hațieganu University of Medicine and Pharmacy Cluj-Napoca, Louis Pasteur Str., no. 6, 400349 Cluj-Napoca, Romania; dana.festila@gmail.com; 6 Clinical Rehabilitation Hospital, Viilor Str., no. 46-50, 400347 Cluj-Napoca, Romania; gbodizs@yahoo.com; 7 Department of Physical Medicine and Rehabilitation, Iuliu Hațieganu University of Medicine and Pharmacy, Louis Pasteur Str., no. 6, 400349 Cluj-Napoca, Romania; dogarugabrielaumf@gmail.com; 8 Department of Patho-Biochemistry, University of Medicine and Pharmacy Craiova, Petru Rareș Str., No. 2-4, 200349 Craiova, Romania; 9 Vascular Neurology and Dementia, University of Medicine, Essen, HufelandStr., no. 55, 45122 Essen, Germany

**Keywords:** oxidative stress, periodontitis, periodontal pocket, ischemic stroke, chronic inflammation

## Abstract

*Background*: Due to the higher frequency of ischemic stroke in men compared to women, we aimed to determine if gender differences exist regarding periodontal status and several plasma biomarkers in patients with a recent large artery atherosclerosis ischemic stroke (IS). *Material and methods*: Patients with their first IS within less than six weeks who were able to undergo periodontal examinations were evaluated. Demographic data, periodontal status, oxidative stress parameters/plasma antioxidant capacity, and C-reactive protein in patients who suffered a recent large artery atherosclerosis ischemic stroke were reccorded. *Results*: 93 patients were included in the study. More men were smokers (12/57 vs. 3/36) and consumed alcohol (17/57 vs. 3/36), and more women had higher glycemic values (*p* = 0.023), total cholesterol (*p* < 0.001), LDL (low-density lipoprotein)-cholesterol (*p* = 0.010), and HDL (high-density lipoprotein)-cholesterol (*p* = 0.005) levels. Significantly more men than women had moderate plus severe periodontal disease (*p* = 0.018), significantly higher levels of nitric oxide (*p* = 0.034), and significantly lower levels of total antioxidant capacity (*p* = 0.028). *Conclusions:* In this pilot study, men seem to be more prone to oxidative stress and to develop more severe forms of periodontitis among patients with stroke, but the results need validation on a larger sample.

## 1. Introduction

Ischemic and hemorrhagic stroke are two of the most common causes of mortality and adult disability worldwide [1,2]. Despite the increased risk for stroke in post-menopausal women, men have higher frequency of stroke even in old age, while the cause of this difference is still under evaluation [3,4,5,6]. Almost 80% of strokes are caused by focal cerebral ischemia, while the remaining occur due to hemorrhages [7]. Atherosclerosis of major intracranial arteries remains one of the primary etiological factors for ischemic stroke, [8] but some risk factors, such as age, gender, hypertension, diabetes mellitus, smoking, and severe tooth loss, have been associated with cerebrovascular disease [9].

Periodontitis (periodontal disease) is a ubiquitous chronic infectious-inflammatory disease [10,11,12,13,14,15]. Periodontitis has a negative impact on oral function, quality of life, and general health. Moreover, severe periodontitis has been independently associated with increased mortality in several different populations [16].

Wu et al. first reported the epidemiological and etiopathogenetic associations between chronic periodontitis and stroke [17], and Straka and Trapezanlidis emphasized the possible relationships between oral infections and stimulation of atherogenic mechanisms [18]. Since then, other studies have reported an association between periodontitis and acute ischemic stroke [17,19,20,21,22,23], while several reviews showed a moderate association between periodontitis and stroke [24,25,26,27,28,29]. However, Khlande et al. found no strong evidence for such an association [30].

Several confounding risk factors for periodontitis and cerebrovascular disease have been highlighted, such as diabetes mellitus, smoking, and age [31]. However, an independent association between periodontal disease and incident stroke risk has been reported [32].

Leira et al. reported a 2.8-fold higher risk of developing ischemic stroke in patients with periodontitis compared to those without periodontitis [24]. In addition, Aarabi et al. claimed that chronic oral infections are associated with an imaging surrogate of cerebrovascular ischemia beyond acute ischemic stroke (e.g., silent infarcts and brain white matter hyperintensities) related to small vessel disease [33]. A dose-dependent association has also been reported between tooth loss as a measure of periodontitis severity and an increased risk for stroke [27,34,35]. It is not clear whether the stroke-periodontitis relationship occurs prior to the stroke or develops later, but due to the long-standing characteristic of periodontitis in which the tissue destruction develops during many years (or decades), it is highly probable that severe alveolysis develops before stroke [36].

Periodontitis could be involved in chronic systemic diseases and the pathogenesis of atherosclerosis via a periodontium-originating low-level bacteremia invading the arterial walls, the release of local inflammatory mediators into the general circulation, autoimmunity to host proteins, and pro-atherogenic effects of bacterial toxins [37,38]. The persistent systemic inflammation triggered by periodontitis induces vascular endothelial dysfunction [25] and may increase inflammation in existing atherosclerotic lesions, thereby increasing the risk of cardiovascular disease and its associated events [37,38]. The concept of the “brain-oral axis” has been introduced to emphasize the impact of oral microbiota on the health of the brain [39,40]. The presence of *Porphyromonas gingivalis*, an important periodontal pathogen, has been linked to an increased risk of ischemic stroke [41], and a targeted periodontal treatment against this bacteria protects against ischemic stroke [42]. 

The presence of a proinflammatory status in patients with periodontitis is associated with the increase in systemic pro-inflammatory mediators, such as C-reactive protein (CRP) [43], and stimulation of the innate and adaptive immune responses.

One of the essential triggers for inflammation is increased oxidative stress and decreased antioxidant molecular activity. Bacteria present in subgingival areas disrupt this balance and could constitute a significant risk factor for stroke [44,45]. Oxidative stress associated with a lower serum total antioxidant level and salivary antioxidant capacity is involved in the progression of periodontitis [46]. Neutrophils and other phagocytes together with other periodontal cells, such as monocytes, gingival fibroblasts, and periodontal ligament cells, enhance production of reactive oxygen species (ROS) upon stimulation by periodontal pathogens and/or their components in *in vitro* studies [47,48].

Altered redox signaling caused by increased bioavailability of ROS is a significant contributor to the onset and/or progression of atherosclerosis and hypertension [49]. ROS include free radicals, such as superoxide and hydroxyl radical, and non-radicals, such as hydrogen peroxide [50]. Oxidative stress induces inflammation that can further augment ROS production, according to Kumar et al. [44]. Oxidative stress plays a major role in endothelial dysfunction and the onset and progression of atherosclerosis. Superoxide can react with nitric oxide (NO) to form peroxynitrite, which reduces the bioavailability of NO that has vasodilatory function [51].

Atherosclerosis and its primary complications, myocardial infarction and stroke, remain a major cause of death and disability worldwide [51]. However, no conclusions can be drawn in the relationship between periodontitis and stroke. If causal, the association would be important because treating periodontitis could potentially reduce the risk for stroke [51]. Well-designed prospective studies should be carried out to provide robust evidence for the association between periodontitis and stroke together with an appropriate adjustment for confounding vascular risk factors and restrictive diagnostic criteria for periodontitis [33]. Considering globally, men continue to have a higher incidence of ischemic stroke than women [6]. In this context, this study evaluated the gender differences regarding periodontal status, oxidative stress parameters/plasma antioxidant capacity, and C-reactive protein in patients who suffered a recent large artery atherosclerosis ischemic stroke.

## 2. Methods

### 2.1. Study Design and Participants

A cohort pilot study was conducted at the Neurology Department, Clinical Rehabilitation Hospital Cluj-Napoca, after receiving ethical approval from the Institutional Ethics Committee (No. 3/2018). Written informed consent was obtained from all participants before they underwent the physical examination, blood sampling, and periodontal examination. This study adhered to principles outlined in the Declaration of Helsinki on experimentation involving human subjects. The study was conducted between May 2018 and February 2019.

Patients aged 18–80 years with ischemic stroke, defined according to the World Health Organization criteria as “the rapid development of localized or global signs of brain dysfunction with symptoms lasting more than 24h without any other apparent cause except those of vascular origin” were eligible for the study [52]. The physical examination (I.S. and A.C.B.) was done to assess the degree of neurological deficit and assess general apprerance and orgnas systems (vital signs, deafness, carotid bruit, urinary incontinence or erectile dysfunction, dysarthia, muscle weekness, vertigo, defness, nystagmus and hemiparesis, gait abnormalities or ataxia, cranial nerve abnormalities). Ischemic stroke was confirmed by a cranial computed tomography (CT) examination. All patients with large artery atherosclerosis stroke (sub-type 1, TOAST classification - Trial of Org 10172 in Acute Stroke Treatment) [53] were included in the study.

According to the hospital register, investigators consecutively recruited cases each day based on the inclusion/exclusion criteria. The patients were referred from other hospitals within the county as well as from the central-northern part of Romania. 

All patients included in this study had a complete neurologic and cardiologic evaluation. Patients at their first ischemic stroke were included if ischemic stroke onset was less than 6 weeks, they were able to undergo a dental examination, and they could provide informed consent. The patients included in the study were undergoing antiplatelet therapy initiated during the acute phase of ischemic stroke and antihypertensive therapy was applied for those who had hypertension. Patients with diabetes mellitus were under treatment with oral hypoglycemic drugs or insulin therapy.

All patients with a hemorrhagic stroke (confirmed by cranial CT examination) or recent myocardial infarction were excluded. Patients with acute infections (requiring antibiotic treatment), inflammatory disorders, degenerative brain diseases, oncologic diseases (in the past 5 years), on immunosuppressant therapy, with periodontal treatment in the last year or with recent (<1 month) history of tooth extraction were also excluded. 

The STROBE (StrengThening the Reporting of Observational Studies in Epidemiology) guidelines were used to ensure accurate reporting of this study [54].

### 2.2. Demographic and General Medical Characteristics Evaluation

Demographic data, such as age, gender, BMI (kg/m^2^), and behavioral risk factors (namely smoking, and alcohol consumption) were recorded. Blood pressure (systolic and diastolic blood pressure), and electrocardiogram were measured in all patients as input data to identify the presence of cardiac co-morbidities.

### 2.3. Assessment of Blood Parameters

Blood samples were taken from all subjects included in this study in the morning after a 12 h fast. The following parameters were measured: basal glycemia, total cholesterol, LDL and HDL-cholesterol, triglycerides, and CRP (a biomarker for inflammation). Oxidative stress was assessed by measuring the oxidative stress biomarkers, such as MDA (malondialdehyde), NOx (the indirect assessment of NO synthesis), total oxidative stress (TOS), and plasma antioxidant status was assessed by measuring the thiol, catalase and total antioxidant capacity (TAC) plasma level, as described previously [55,56].The plasma parameters of oxidative stress/antioxidant capacity were assessed using a spectrophotometer (Jasco International Co., Ltd., Tokyo, Japan). MDA assessment was made by using thiobarbituric acid reaction, reported as a standard method [57,58,59]. Briefly, the serum sample was mixed with trichloracetic acid and vigorously vortexed, then thiobarbituric acid was added. After the solution was kept for 30 min at 95 °C followed by ice cooling and centrifugation, the absorbance of each sample was determined at 530 nm compared with a blank [55,56]. The NOx was assessed by Griess reaction (reduction of nitrate to nitrite by mixing the plasma sample with vanadium chloride and further mixing with Griess reagent, followed by sample absorbance measurement at 540 nm [55,56]). TOS was determined by Erel method: plasma was added to Erel reagent, vortexed, incubated for 30 min at room temperature and followed by absorbance measurement at 560 nm [60]. TAC assessment was made also by a method described by Erel: plasma was mixed with Erel reactive, kept for 10 min at room temperature, followed by absorbance determination at 444 nm [60]. Catalase was assessed by the method described by Aebi [61] that measure the catalase activity from decomposition of hydrogen peroxide. Thiols assessment was made by Ellman method [62]: a buffer solution was added to plasma sample, followed by mixing with 3,5-dinitrobenzoic acid and incubation at room temperature for 15 min. The sample’s absorbance was measured by spectroscopic method at 417 nm.

All chemicals were obtained from Sigma-Aldrich Co. (St. Louis, MO, USA). All substances used were of analytical grade.

### 2.4. Periodontal Examination

Periodontal measurements and recordings were performed by two previously calibrated, experienced investigators (I.C.M. and D.G.F.), filling for each patient using the *Periodontal Chart* (see Supplementary Materials). Further, both investigators attended two training meetings, in the presence of a senior periodontist, where they received oral and written instructions on development of the study examination protocol [63] and were given data compilation sheets and their precise role and responsibilities in the study. 

A full-mouth periodontal examination in a standard environment using standard methodology and equipment was applied to all patients. The oral health screening was conducted in natural light using a dental mirror and a 1 mm marking periodontal probe (UNC-15 periodontal probe, Hu-Friedy, Chicago, IL, USA). Six sites per tooth were evaluated for probing depth (PD), gingival recession (GR), and clinical attachment loss (CAL) [14,64]. PD, GR, and CAL were evaluated according to standard clinical definitions. All probing measurements were rounded down to the nearest millimeter. 

The full-mouth Gingival Bleeding Index (GBI) was calculated. The GBI was defined as the total number of sites with gingival bleeding on probing divided by the total number of sites per mouth (four sites at each tooth) multiplied by 100 [65]. The number of missing teeth was also recorded.

Periodontal status was defined initially as a five-level categorical variable according to the previously published definition [14,66,67]: severe periodontitis, moderate periodontitis, mild periodontitis, gingivitis, or periodontal health. The Center for Disease Control and the American Academy of Periodontology case definition of periodontitis was based on measures of CAL and PD at interproximal sites [14]. Severe periodontitis was defined as having at least 2 sites with CAL ≥6 mm (not on the same tooth) and at least 1 interproximal site with PD ≥5 mm. Moderate periodontitis was defined as 2 or more interproximal sites with CAL ≥4 mm (not on the same tooth) or 2 or more interproximal sites with PD ≥5 mm, also not on the same tooth. Mild periodontitis was defined as at least 2 interproximal sites with CAL ≥3 mm and at least 2 interproximal sites with PD ≥4 mm (not on the same tooth) or 1 site with PD ≥5 mm. Gingivitis patients were cases that did not meet the preceding definitions and had a GBI >10% and probing depths ≤3 mm. The remaining subjects were considered to have healthy periodontal tissue [67].

### 2.5. Data Analysis

The five-level periodontal categorical variables were regrouped as healthy periodontium or gingivitis, mild periodontitis, and moderate or severe periodontitis for the statistical analysis. The grouping aimed to reflect the intensity of the inflammatory local burden, which increased with the severity of the periodontal disease. Periodontitis was considered when mild, moderate, or severe periodontitis existed.

Quantitative data were tested for normality on sub-groups with the Shapiro–Wilk test (for groups lower than 30 patients) or Kolmogorov-Smirnov test (groups larger than 29 patients) according to the sample size of the group. The values are reported as mean (standard deviation) when they followed a normal distribution. Otherwise, the quantitative data were reported as median (Q1–Q3), where Q is the quartile (first and third, respectively). The categorical data are reported as absolute and relative frequencies. The following statistical tests were used to compare groups: Student’s t-test for independent samples (quantitative data normally distributed, two groups), the Mann–Whitney test (nonnormally distributed quantitative data, two groups), Chi-square or Fisher exact test (categorical data), and the Kruskal–Wallis test (nonnormally distributed quantitative data for more than two groups). The Z-test for proportions was also used to compare categorical data between women and men. The Statistica program (v. 8; StatSoft, Tulsa, OK, USA) was used to analyze the data. A *p*-value < 0.05 was considered statistically significant whenever two groups were compared, and has been adjusted by the number of groups whenever more than two groups were compared (*p*-value < 0.013 for the Kruskal–Wallis test).

## 3. Results

**Figure 1:**
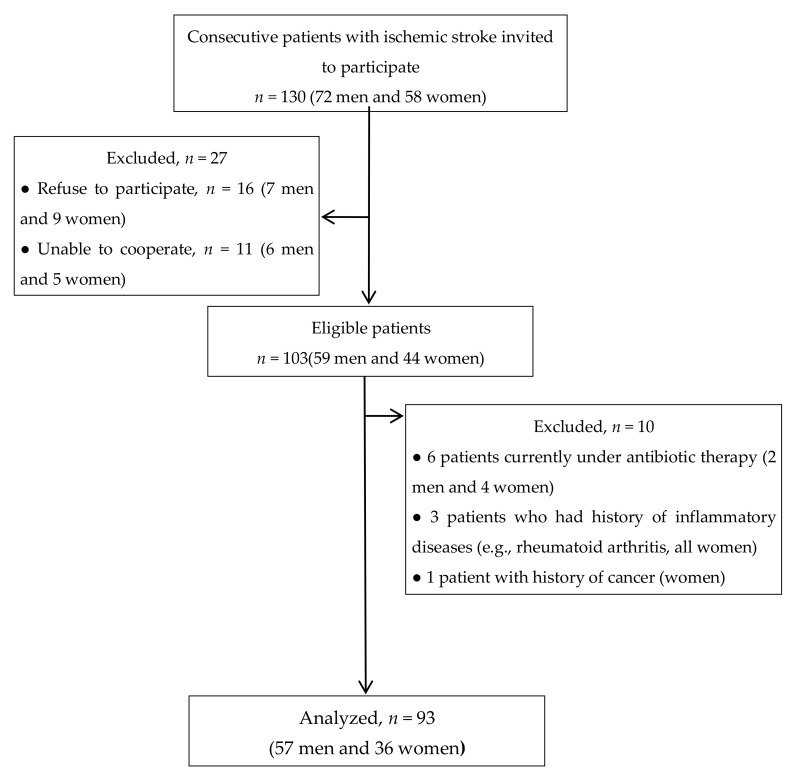
Flow chart of the patients.

Ninety-three patients with ischemic stroke (age 44–80 years) satisfied the inclusion criteria and agreed to participate in the study (Figure 1). The patients’ demographic and clinical characteristics and behaviors are summarized in Table 1. More frequently, the men were current smokers than women (12/57, 21.1% vs. 3/36, 8.3%) and consumed alcohol (17/57, 39.8% vs. 3/36, 8.3%). Most of the patients were overweight (53.8%) or obese (14.0%), but the frequencies were not significantly different between genders (*p* = 0.185). 

Abnormal blood glucose levels were observed in 25 patients (26.9%), without a significant difference between the genders (*p* = 0.111). Sixteen patients (10 women and 6 men) had total cholesterol levels outside the normal ranges (women:men = 27.8%:10.5%; *p* = 0.032). In contrast, more men had HDL-cholesterol levels outside the normal ranges compared to women (men:women = 21.1%:2.8%; *p* = 0.013).

The blood levels of all investigated oxidative stress and antioxidants biomarkers were outside the normal ranges except total antioxidant capacity. The total antioxidant capacity values were in normal range of 15.1% of the subjects, without a significant difference between genders (men:women = 12.3%:19.4%, *p* = 0.347). Higher levels of serum NOx were observed in men and higher serum levels of TAC were detected in women (see Table 2) without other significant differences in the oxidative stress and antioxidant biomarkers.

More women (11/36) than men (8/57) were edentulous (*p* = 0.054), but more men (43/49) than women (21/25) had periodontal disease (*p* = 0.331). 

No significant association was observed between periodontal disease and gender (*p* = 0.079, Table 2), but the frequency of men with ischemic stroke and moderate plus severe periodontal disease (56.1%) was significantly higher than the percentage of women with stroke and moderate or severe periodontal disease (30.6%) (Z-test for two proportions; *p* = 0.018).

No significant differences were observed when the investigated oxidative stress and antioxidant biomarkers were compared according to periodontal disease at an adjusted significance level of 0.013 (Table 3). Comparable distributions of smokers (ranging from 2/19 in the edentulous group to 2/21 in the mild periodontitis group) and alcohol consumption (ranging from 1/10 in the healthy periodontium and gingivitis group to 5/21 in the mild periodontitis group) were observed among the periodontal disease groups. Similar distribution among periodontal disease groups were observed regarding the presence of hypertension (from 17/21 in the mild periodontitis group to 18/19 in the edentulous group) and ischemic heart disease (from 3/10 in the healthy periodontium and gingivitis group to 11/21 in the mild periodontitis group). The majority of patients in the healthy periodontium and gingivitis group had diabetes mellitus (8/10), while the presence of this co-morbidity had similar distribution among those with mild periodontitis (6/21) and moderate and severe periodontitis (13/43). 

## 4. Discussion

In our group of stroke patients, an association tended to be observed between periodontal disease and gender (*p* < 0.10), with significantly more men with moderate or severe periodontitis than women (*p* < 0.02). The oxidative stress and antioxidant parameters were similar among the different stages of periodontal disease. NOx as an oxidative stress parameter and TAC as an antioxidant parameter were significantly different between the genders in patients with ischemic stroke, but the small differences could also be by chance and thus need caution in the interpretation and further validation.

The sample was comprised of more men than women (Table 1), which is an expected result associated with the characteristics of the primary disease represented by stroke [68]. The age of the evaluated patients was similar between men and women but women had a tendency to have a significantly higher body mass index (BMI) than men (*p* < 0.10). Overweight was more frequently observed in our sample (53.8%) compared to the values reported previously in stroke cohorts (44.2% in the community-based Framingham Heart Study) [69]. The higher BMI values in women than men (Table 1) could be attributed to difficulties in controlling weight during pregnancy, and hormonal changes during the postmenopausal period [70] as well as an increased frequency of subclinical hypothyroidism [71]. Perissinotto et al. also reported a significantly higher BMI than men, suggesting that visceral redistribution in old age predominantly affects more women than men [72]. A higher BMI in women than men is associated with a significant increase in all lipid metabolic parameters in women than men [73], and the link between these pathogenetic loops is represented by disturbances in thyroid function that can influence lipid metabolism [71,74].

Our results show a significant increase in total cholesterol, LDL-CST, and TGs in women than men and also a significant decrease in HDL-CST in men than women (Table 1). It has been suggested that periodontitis is significantly associated with lower HDL-CST levels and respective increases in LDL-CST and triglyceride levels, supporting the hypothesis that periodontitis is associated with disturbances in the control of lipid metabolism [75].

In our study, smoking and alcohol consumption were predominant in men, but the results could not be validated statistically due to the small sample. According to previous studies, 18.7% of stroke survivors are active smokers, despite strong recommendations to stop smoking, and 51.7% are men [76]. 

The assessment of the association between periodontitis and smoking or alcohol consumption was not statistically evaluated in our study because the sub-groups were very small. Current smokers have an increased prevalence, severity, and progression of periodontitis as well as a negative therapeutic response compared to never or former smokers [77]. Quitting smoking limits the destruction of the periodontal tissues and leads to better clinical results [78]. Alcohol consumption is also more frequent in men who are stroke survivors compared to women [79,80,81], as in our study. The risk for periodontitis among men reporting regular alcohol intake previously proved dose-dependency [82]. 

Alcohol consumption impairs neutrophil, macrophage, and T-cell functions, increasing the likelihood of connective tissue inflammation and stimulating alveolar bone resorption [83]. 

No significant differences between genders were found regarding the number of patients with hypertension or ischemic heart disease (Table 1). Women from the study group had a higher frequency of diabetes mellitus than men, which was directly related to significantly higher basal glycemia (Table 1). The frequency of diabetes mellitus between women and men varies according to the global region but is estimated to have a complex relationship with differences in ethnicity, migration, culture, lifestyle, gene-environment interactions, socioeconomic status, and social roles [84]. The reported difference could also be explained by random testing, selection bias, and differences in access to healthcare in some countries [85,86,87]. The difference could also be a result of the insulin resistance as an incipient phase of type 2 diabetes mellitus [88,89]. Our study found the highest frequency of diabetes mellitus among patients in the healthy periodontium and gingivitis group that could be explained by the healthcare awareness of these patients. Diabetes mellitus is a known risk factor for the development, progression, and increasing severity of periodontitis [90]. Immune dysfunction in diabetes due to unresolved infection with opportunistic microorganisms is one of the pathways driving periodontitis. Advanced glycated end products are involved in destroying periodontal tissues through enhanced inflammation, impaired wound repair, and increased oxidative stress [91]. The presence of dyslipidemia in diabetic patients is associated with increased destruction of periodontal tissues and gingival inflammation [92] probably due to the stimulation of pro-inflammatory status, which increases the severity of periodontitis [93], or as a source for ROS production in the periodontium through lipid peroxidation [90].

Inflammatory markers (CRP) increased in our study in both men and women (Table 1) with a tendency for significantly higher values in women than men (*p* < 0.10). Elevated levels of CRP are a risk factor for stroke. High CRP values have been reported in patients who suffer an ischemic stroke, and are correlated with prognosis, emphasizing the role of inflammation in stroke pathogenesis [94]. Moreover, CRP, as a biomarker of systemic inflammation, reflects chronic inflammatory status due to periodontitis, and treating a periodontal infection can significantly lower serum CRP levels [95]. These findings support the hypothesis that chronic oral infections and periodontitis-related inflammation are contributors to the systemic inflammatory response [96]. CRP is an acute-phase protein and a biomarker of systemic inflammation produced by the liver in response to various inflammatory stimuli that can trigger its synthesis due to excessive production of pro-inflammatory cytokines, such as interleukin-6 (IL)-6, IL-1β, and tumor necrosis factor-α [97]. The elevated inflammatory factors increase inflammatory activity in atherosclerotic lesions and potentially increase the risk for cardiovascular and cerebrovascular events [98]. Slade et al. reported that patients with extensive periodontal disease (>10% of sites with periodontal pockets >4 mm) have significantly increased CRP levels compared with healthy people [99]. Chronic periodontitis has been associated with elevated CRP levels, even after controlling for several potential confounders, such as age, education level, gender, smoking, HDL-cholesterol, and diabetes [100]. However, in our study CRP values were not associated with periodontitis (Table 3).

Oxidative stress is a mechanism involved equally in the pathogenesis of ischemic stroke and inflammatory diseases, such as periodontitis, which alters the balance between free radical production and plasma antioxidants [101]. Oxidative stress molecules are involved in triggering and enhancing inflammatory reactions [102]. Patients with moderate and severe periodontitis have lower antioxidant capacity and higher oxidative stress markers, such as MDA and NOx [103]. The increase of NOx in patients with periodontitis is explained by increased production of NOx due to activation of inducible nitric oxide synthase (iNOS) by proinflammatory cytokines produced by inflammatory cells at the site of gingival inflammation [104,105]. Increased iNOS has been reported in gingival tissue in patients with periodontitis compared with control subjects [106]. The severity of periodontitisis was also related to salivary nitrite concentration [107]. An increase in serum NOx levels was reported in patients with periodontitis by Menaka et al. [108]. In our study, NOx levels increased significantly in men compared with women (Table 2). This difference could be related to significant increases in smoking among men or to increased alcohol consumption, but this needs validation in a larger cohort.

All serum antioxidant biomarker levels were reduced in men compared with women; only TAC was significantly lower in men than women (Table 2, *p* < 0.03). One of the most critical antioxidant molecules that contribute to the antioxidant defense mechanism is represented by catalase. Despite the fact in our study catalase was not significantly statistically different in men compared to women, catalase was previously demonstrated to be lower in gingival tissues in patients with chronic periodontitis, and a greater decrease occurs within tissues adjacent to pockets deeper than 6 mm [109]. Our study also demonstrated that men suffered from a higher frequency of moderate-to-severe periodontitisthan women (*p* < 0.02). Duarte et al. [110] reported that gene expression of catalase is upregulated in poorly controlled diabetics with chronic periodontitis. The increased catalase activity in patients with diabetes has been attributed to protective and adaptive mechanisms that develop in tissues in response to oxidative stress [111]. In the present study, women had significantly higher basal glycemia compared with men (Table 1), which supports the hypothesis that the increased catalase in women is the result of an adaptive mechanism to the oxidative stress enhanced by hyperglycemia. 

In our study, more women who suffered a recent ischemic stroke were edentulous than men. Tooth loss in women is associated with a higher risk of all-cause mortality [112]. Tooth loss lacks specificity as a marker of periodontitis because tooth loss occurs not only in the final stage of periodontitis but for many other reasons [83]. However, tooth loss is considered a surrogate indicator of periodontitis, as it occurs in the final stages of the disease and could reflect the persistence of a severe oral inflammation and of a low-grade systemic inflammation. It appears that missing teeth may be a contributing factor determining cardiovascular risk [113]. A significant association has been found between levels of tooth loss and subclinical atherosclerosis, such as the incidence of carotid plaque [114]. Patients with fewer teeth may have an increased risk of stroke [27,35]. A consistently strong and dose-dependent association has been found between the number of missing teeth and incident ischemic stroke and mortality in a large population-based cohort of 4,440,970 relatively young subjects (mean age, 41.5 years) after multivariate adjustment for cardiovascular risk factors [34]. Other studies show no association between missing teeth and stroke [113]. Tooth loss is also related to a lower socioeconomic status in Europe, as poor oral hygiene is a major contributor to periodontal disease and thus a potentially modifiable stroke risk factor. An increase in tooth brushing frequency decreases the concentration of systemic inflammatory markers in serum [115], and dental prophylaxis or periodontitis treatment reduces the incidence of ischemic stroke [42]. Intensive periodontal treatment improves systemic inflammation, high blood pressure, the lipid profile [116], and endothelial dysfunction [117]. An independent role of regular dental care in the prevention of ischemic stroke in an elderly population was reported by Sen et al. [32]. These results indicate that identifying and treating periodontitis at a large scale population level, particularly in high-risk patients, may reduce stroke risk.

This study has some potential limitations. The main limitation is related to sample size and the limited number of patients by gender in each periodontitis group. The small sample size of our pilot study could have hidden some significant differences and also limited the statistical analysis as well as the evaluation of oxidative stress and antioxidant parameters between genders according to the class of periodontal disease. Moreover, no evaluation of the former smoking status, smoking history (e.g., ex-smoker, type: cigarettes, cigars, pipe, chewing, snuff) or smoking quantification (e.g., no. of years, how frequent per day, etc.) was done in the evaluation sample. Smoking history of the patients could bring important information due to the cumulative effect of smoking on both atherosclerosis and periodontitis. Furthermore, the applied experimental design does not allow the assessment of the causality between periodontitis and stroke. However, this pilot study constitutes a starting point for more studies on larger samples with further investigations of NOx, total oxidant status, and total antioxidant capacity. The results reported in this study could be used to calculate the required sample size for further studies carried out of the Romanian population after the evaluation of the parameters on matched controls not affected by ischemic stroke and/or atherosclerosis. Further larger studies investigating the relationships between ischemic stroke subtypes and periodontitis severity are needed to identify the influence of periodontal disease in stroke pathophysiology. However, in the context of actual disastrous epidemiological circumstances, it seems improbable to conduct large-scale clinical studies in the next short time period.

This is the first study to address Romanian stroke patients and observe the impact of periodontitis and oxidative stress markers on stroke. Specialists and authorities seem to have no concerns regarding the systemic impact of periodontitis. The present data could contribute to elaborating large-scale screening and preventive plans after validation of the results on larger samples.

## 5. Conclusions

Serum oxidative stress parameters, such as NOx, constitute important biomarkers indicating the persistence of chronic inflammation in patients with stroke and associated periodontitis, which was significantly higher in men than women in the present study, but the difference was small. This result could be related to the higher frequencies of smokers and chronic alcohol drinkers in men than women. The decreased TAC level in men who suffered an ischemic stroke and associated periodontitis compared with women demonstrates that plasma antioxidative capacity is slightly lower in men than women. The oxidative stress/antioxidant status differences between men and women could contribute to increased severity of periodontitis among men, which could lead to a greater impact on stroke morbidity, but the results need validation on larger samples. Men with severe or moderate periodontitis should alert physicians to conduct extensive examinations, particularly when other stroke risk factors are present. Sex disparities related to stroke and periodontal disease could have an important clinical implication that can indicate resources needed for national public health programs, but the results observed in our study need to be validated on a larger sample and in the context of comparison with a matched control group without ischemic stroke.

## Figures and Tables

**Table 1:** Sample baseline characteristics, grouped by gender.

Characteristics	All, n = 93	Men, n = 57	Women, n = 36	*p* Value
***Demographic***				
Age (years) *	66.4 (10.4)	66.3 (11.0)	66.6 (9.5)	0.908
BMI (kg/m^2^) *	26.8 (3.3)	26.3 (2.7)	27.5 (3.9)	0.076
***Co-morbidities***				
Hypertension, yes **	81 (87.1)	48 (84.2)	33 (91.7)	0.296
Ischemic heart disease, yes **	36 (38.7)	22 (38.6)	14 (38.9)	0.978
Diabetes mellitus, yes **	36 (38.7)	18 (31.6)	18 (50.0)	0.076
***Biochemical parameters***				
Basal glycemia (mg/dL) *	113.5 (21.5)	109.5 (18.6)	119.9 (24.5)	0.023
Total cholesterol (mg/dL) *	177.0 (41.9)	163.9 (40.0)	197.8 (36.5)	< 0.001
LDL-CST (mg/dL) *	107.1 (39.1)	98.8 (34.8)	120.1 (42.4)	0.010
HDL-CST (mg/dl) ***	44.0 (39.0 to 55.0)	42.0 (36.0 to 42.0)	51.0 (41.5 to 59.5)	0.005
TG(mg/dL) ***	117.0 (86.0 to 150.0)	106.0 (84.0 to 106.0)	128.5 (96.3 to 167.0)	0.043
CRP (mg/L) *	2.9 (1.1)	2.7 (1.1)	3.1 (1.1)	0.082

* Continuous data are summarized as mean (standard deviation) or median (*** first to the third quartile). ** Categorical data are summarized as the number of cases (percentages). *p*-values reflect the comparison between men and women: Student’s *t*-test for independent samples was used when the results are reported as means (standard deviation); the Mann–Whitney test was used for data reported as medians (first quartile to third quartile); or the chi-square test was used for categorical data. BMI = body mass index, LDL-CST = low-density lipoprotein cholesterol, HDL-CST = high-density lipoprotein cholesterol, TG = triglycerides, CRP = C-reactive protein

**Table 2:** Oxidative stress biomarkers and periodontal disease-related characteristics.

Characteristics	All, n = 93	Men, n = 57	Women, n = 36	*p* Value
*Oxidative stress* *				
MDA (nmol/mL)	6.6 (0.5)	6.6 (0.5)	6.7 (0.5)	0.529
NOx (μmol/L)	69.3 (3.9) 69.9 (65.7 to 71.4)	70.0 (3.7) 70.5 (67.9 to 72.4)	68.3 (4.0) 68.1 (65.5 to 70.8)	0.034 0.032 **
TOS (μmol/L)	32.5 (2.6)	32.5 (2.6)	32.6 (2.4)	0.926
*Antioxidants* *				
Thiol(μmol/L)	252.9 (18.2)	251.2 (18.5)	255.6 (17.7)	0.258
TAC (mEq/L)	0.95 (0.18) 0.96 (0.80 to 1.08)	0.92 (0.17) 0.95 (0.78 to 1.02)	1.01 (0.18) 0.98 (0.86 to 1.13)	0.028 0.046 **
Catalase (U/mL)	65.0 (5.3)	64.9 (5.5)	65.0 (5.2)	0.912
*Periodontal related parameters* **				
Gingival Bleeding Index (GBI), %	52.5 (33.0 to 77.0)	50.0 (33.0 to 50.0)	64.6 (36.5 to 87.9)	0.062
Missing teeth	20 (15 to 29)	20 (13 to 28)	23 (16 to 31)	0.079
Periodontal disease *** Edentulous Healthy periodontium plus gingivitis Mild periodontitis Moderate plus severe periodontitis	19 (20.4) 10 (10.8) 21 (22.6) 43 (46.2)	8 (14.0) 6 (10.5) 11 (19.3) 32 (56.1)	11 (30.6) 4 (11.1) 10 (27.8) 11 (30.6)	0.079

Continuous data are summarized as mean (standard deviation) * or median (first to third quartiles) ** Categorical data are summarized as the number of cases (percentages) *** *p*-values reflect the comparisons between men and women; Student’s *t*-test was used for independent samples when results are reported as means (standard deviation) (*); the Mann–Whitney test was used for data reported as medians (first to third quartiles) (**); the chi-square or Fisher exact test was used for categorical data (according to the expected values) (***). PD = periodontal probing depth; CAL = clinical attachment loss; MDA = malondialdehyde; NOx = indirectly assessed nitric oxide; TOS = total oxidative stress; TAC = total antioxidant capacity.

**Table 3:** Differences among the periodontal disease groups.

.	Edentulous n = 19	Healthy Periodontium and Gingivitis n = 10	Mild Periodontitis n = 21	Moderate and Severe Periodontitis n = 43	*p* Value
Age, years	74 (65 to 80.5)	65 (59 to 74.8)	64 (58 to 68)	65 (59.5 to 70)	0.037 *
Basal glycemia (mg/dL)	112 (99.5 to 123.5)	136.5 (110.8 to 143.5)	106 (97 to 117)	106 (95.5 to 124)	0.091 *
CRP (mg/L)	2.9 (2.2 to 3.9)	3.8 (2.8 to 4.4)	3.0 (2.4 to 3.1)	2.5 (1.9 to 3.4)	0.120 *
MDA (nmol/mL)	6.5 (6.3 to 7.0)	6.9 (6.2 to 7.1)	6.5 (6.3 to 7.1)	6.7 (6.3 to 7.0)	0.952 *
NOx (μmol/L)	70.6 (68.4 to 71.3)	70.7 (68.8 to 73.0)	66.4 (65.5 to 69.3)	70.3 (65.7 to 72.34)	0.050 *
TOS (μmol/L)	32.2 (31.2 to 34.1)	35.2 (33.2 to 36.7)	31.1 (30.5 to 33.1)	32.3 (30.5 to 33.6)	0.055 *
Thiol (μmol/L)	245 (242 to 265.5)	245.0 (228 to 262.8)	257 (243 to 267)	254 (239 to 267)	0.557 *
TAC (mEq/L)	1.0 (0.8 to 1.1)	0.9 (0.8 to 1.0)	1.1 (1.0 to 1.1)	1.0 (0.8 to 1.0)	0.038 *
Catalase (U/mL)	64.3 (60.4 to 67.8)	65.3 (59.5 to 69.8)	66.2 (61.2 to 71.2)	65.7 (61.7 to 68.1)	0.728 *

Continuous data are summarized as median (first to third quartiles) * *p*-values reflect the comparisons between the groups with the Kruskal-Wallis test. MDA = malondialdehyde; NOx = nitric oxide; TOS = total oxidative stress; TAC = total antioxidant capacity.

## Supplementary Materials

The following are available online at https://www.mdpi.com/2077-0383/9/6/1744/s1, Figure S1: Periodontal Chart.
