# Supplementary material for: Gender Differences in the Levels of Periodontal Destruction, Behavioral Risk Factors and Systemic Oxidative Stress in Ischemic Stroke Patients: A Cohort Pilot Study
Source: J Clin Med. 2020 Jun 4;9(6):1744. doi: 10.3390/jcm9061744 (PMC7356570; doi:10.3390/jcm9061744)
Supplement: Supplementary file 1 [file jcm-09-01744-s001.pdf]

Journal of
Clinical Medicine

MDPI

Supplementary material: Periodontal Chart

18	17	16	15	14	13	12	11	21	22	23	24	25	26	27	28
GBI score: ......%															
48	47	46	45	44	43	42	41	32	32	33	34	35	36	37	38

Periodontal Chart

Date:

Patient ID:

Age:

		MB[4]	CB[5]	DB[6]	DO[7]	CO[8]	MO[9]	Furc[10]	Mob[11]
**18**	PD[1]								
	GR[2]								
	CAL[3]								
**17**	PD								
	GR								
	CAL								
**16**	PD								
	GR								
	CAL								
**15**	PD								
	GR								
	CAL								
**14**	PD								
	GR								
	CAL								
**13**	PD								
	GR								
	CAL								
**12**	PD								
	GR								
	CAL								
**11**	PD								
	GR								
	CAL								
**21**	PD								
	GR								
	CAL								
**22**	PD								
	GR								
	CAL								
**23**	PD								
	GR								
	CAL								
**24**	PD								
	GR								
	CAL								
**25**	PD								
	GR								
	CAL								
**26**	PD								
	GR								
	CAL								
**27**	PD								
	GR								
	CAL								
**28**	PD								
	GR								
	CAL								

		MB	CB	DB	DO	CO	MO	Furc	Mob
**38**	PD								
	GR								
	CAL								
**37**	PD								
	GR								
	CAL								
**36**	PD								
	GR								
	CAL								
**35**	PD								
	GR								
	CAL								
**3**	PD								
	GR								
	CAL								
**33**	PD								
	GR								
	CAL								
**32**	PD								
	GR								
	CAL								
**31**	PD								
	GR								
	CAL								
**41**	PD								
	GR								
	CAL								
**42**	PD								
	GR								
	CAL								
**43**	PD								
	GR								
	CAL								
**44**	PD								
	GR								
	CAL								
**45**	PD								
	GR								
	CAL								
**46**	PD								
	GR								
	CAL								
**47**	PD								
	GR								
	CAL								
**48**	PD								
	GR								
	CAL								

Legend: [1] Pocket Depth; [2] Gingival Recession; [3] Clinical Attachment Loss; [4] Mesio-Buccal; [5] Centro-Buccal; [6] Disto-Buccal; [7] Disto-Oral; [8] Centro-Oral; [9] Mesio-Oral; [10] Furcation Lesion; [11] Tooth Mobility
